# Safety and Efficacy of Transarterial Chemoembolization Combined With Immune Checkpoint Inhibitors and Tyrosine Kinase Inhibitors for Hepatocellular Carcinoma

**DOI:** 10.3389/fonc.2021.657512

**Published:** 2022-01-13

**Authors:** Fei Yang, Jun Yang, Wei Xiang, Bin-Yan Zhong, Wan-Ci Li, Jian Shen, Shuai Zhang, Yu Yin, Hong-Peng Sun, Wan-Sheng Wang, Xiao-Li Zhu

**Affiliations:** ^1^ Department of Interventional Radiology, The First Affiliated Hospital of Soochow University, Suzhou, China; ^2^ Department of Interventional Radiology, Affiliated Hospital of Jiangnan University, Wuxi, China; ^3^ Department of Oncology Intervention, Taizhou Municipal Hospital, Taizhou, China; ^4^ Department of Child Health, Jiangsu Key Laboratory of Preventive and Translational Medicine for Geriatric Diseases, School of Public Health, Soochow University, Suzhou, China

**Keywords:** hepatocellular carcinoma, tyrosine kinase inhibitors, immune checkpoint inhibitors, TACE, immunotherapy, targeted therapy

## Abstract

**Purpose:**

To explore the safety and efficacy of transarterial chemoembolization (TACE) in combination with immune checkpoint inhibitors (ICIs) and tyrosine kinase inhibitors (TKIs) for the treatment of unresectable hepatocellular carcinoma (uHCC).

**Materials and Methods:**

From August 2019 to July 2020, patients who received TACE combined with ICIs and TKIs were retrospectively analyzed. Treatment-related adverse events (AEs) were recorded. The Kaplan–Meier method was used to estimate time to progression (TTP) and progression-free survival (PFS).

**Results:**

In total, 31 patients with uHCC were included. Eleven patients were classified as BCLC-C. Nineteen patients had multiple lesions, and the cumulative targeted lesions were 69 mm (range, 21-170 mm) according to mRECIST. Twenty-nine (93%) patients experienced at least one AE during the treatment. Four (12.9%) patients developed AEs of higher grade (grade≥3). The objective response rate (ORR) and disease control rate (DCR) were 64.5% and 77.4%, respectively. The median time to response was 7 weeks (range, 4-30 w), and the duration of response was 17.5 weeks (range, 2-46 w). From the first ICIs, TTP and PFS were 6.5 months (95% CI, 3.5-11) and 8.5 months (95% CI, 3.5-NE), respectively.

**Conclusions:**

TACE combined with ICIs and TKIs shows an acceptable safety profile and considerable efficacy in patients with HCC.

## Introduction

Despite surveillance for high-risk patients, hepatocellular carcinoma (HCC) is often diagnosed at intermediate or advanced stages and is not suitable for curative treatment. For these patients, the main treatments are transarterial chemoembolization (TACE) and systemic therapy, such as tyrosine kinase inhibitors (TKIs) and immune checkpoint inhibitors (ICIs) (1).

TACE is the standard of care for unresectable hepatocellular carcinoma (uHCC). In carefully selected patients, the median overall survival (mOS) can reach 20-45 months (2, 3). Nevertheless, TACE remains a palliative therapy, partly because TACE can aggravate hypoxia of residual viable tumors, which further leads to an immunosuppressive microenvironment by overexpression of VEGF, upregulating PD-L1 expression and inhibiting T cell function (4, 5). At the same time, locoregional therapies, including TACE, have been proven to release abundant tumor antigens and have potential synergistic effects with ICIs (6). These findings have aroused interest in further research on the combination of TACE with TKIs or ICIs. Several studies have investigated combining TACE with TKIs (targeting angiogenesis), and most have reported longer time to progression (TTP) but no OS benefits (7, 8). Recently, two small nonrandomized studies tested combining TACE with ICIs and reported relatively promising results (9, 10).

Immune checkpoint inhibitor (ICIs) immunotherapy for HCC is a rapidly developing area (11). Recently, camrelizumab (anti-PD-1 antibody) received approval as the second-line therapy for uHCC in China (12). The objective response rate (ORR) of camrelizumab in uHCC was 14.9%; when combined with apatinib (anti-VEGFR2 antibody), an ORR of 50% was reported (13). This synergistic effect of ICIs and TKIs was confirmed by an RCT study, IMbrave 150 (14), wherein atezolizumab (anti-PD-L1) plus bevacizumab (anti-VEGF antibody) achieved an ORR of 33% and a disease control rate (DCR) of 73.6%. Compared with the sorafenib arm, the risk of death was reduced by 42% (HR:0.58, 95%CI:0.42-0.79; P<0.001) (14), becoming the only first-line therapeutic schedule to outperform sorafenib. Recently, IMbrave 150 group have reported their updated OS analysis, mOS was 19.2 months with atezolizumab +bevacizumab vs 13.4 months with sorafenib(HR,0.66, 95%CI:0.52-0.85; P=0.0009) (15).

Antiangiogenic treatment, such as TKIs, promotes the infiltration of effector lymphocyte cells into tumor microenvironment(TME) through “tumor vascular normalization”, meanwhile alleviates hypoxia and reduces immunosuppression, therefore enhances the efficacy of immunotherapy (16). ICIs may restore the immune-supportive TME through inhibiting immune checkpoints and promoting vascular normalization (17). Thus, TKIs plus ICIs develop a positive reinforcing feedback loop to deal with the hypoxic and immunosuppressive TME. As mentioned above, TACE can exacerbate the hypoxic and immunosuppressive microenvironment of residual tumors. Therefore, the combination of ICIs and TKIs appeared to be a promising strategy to deal with this situation. It is sensical to combine TACE with ICIs and TKIs. In fact, the only case reports in this area have shown a significant response (18). The benefits and risks of this triple combination therapy for uHCC need to be confirmed by further study. Thus, this study investigated a group of patients with uHCC who received TACE with ICIs and TKIs.

## Materials and Methods

This single-center retrospective study was approved by the Institutional Review Board of the First Affiliated Hospital of Soochow University (Suzhou, Jiangsu Province, China), and written consent was obtained from every patient. Patients were diagnosed according to ESMO guidelines (19), and HCC was established based on typical imaging features (enhancement of liver lesions in the arterial phase with washout in the portal venous phase) and/or biopsy or previous surgical resection. Patients concurrently treated with TACE and ICIs (anti-PD-1, camrelizumab) and TKIs (sorafenib or lenvatinib) were included in this study. TACE and ICIs within three months (9) and TACE and TKIs within one month (7, 20) were defined as combination therapy of TACE+ICIs+TKIs. Other key inclusion criteria included (1)18-80 years old,(2)Eastern Cooperative Oncology Group performance status(ECOG-PS) 0-1,(3) Child-Pugh class A or B. Exclusion criteria were as follows:(1)with other malignant tumors,(2)targeted lesion received other locoregional therapy after received ICIs, such as ablation, Brachytherapy, etc. (3) without at least one follow-up image data.

Decisions on the therapeutic schedule were made using a multidisciplinary treatment model (MDT). TACE was routinely performed to control tumor burden for unresectable HCC with preserved liver function. After informing the patients of the costs, possible risks and benefits, the doctor and the patients jointly decided whether to receive ICIs and/or TKIs.

### TACE Procedure

TACE was performed in a superselective fashion. Intraprocedural cone beam CT (CBCT) was used to identify tumor feeding arteries. A 2.3 Fr microcatheter was used to catheterize these tumor feeding arteries. After confirmation by CBCT, emulsions of lipiodol and chemotherapeutic agent (THP10-20 mg) were injected into tumor feeding arteries. Lipiodol consumption was less than 20 ml. If necessary, additional embolization with gelatin sponge or polyvinyl alcohol particles (300-500 μm) was conducted until there was no tumor staining. The TACE procedure was performed by 3 different operators with a median of 15 years of experience. Repeat TACE was performed on a demand model.

### Systematic Therapy

Camrelizumab was given at a dose of 200 mg every 3 weeks intravenously. TKIs were orally administered at an initial daily dose of 800 mg for sorafenib and 8 mg or 12 mg for lenvatinib. Fixed-dose administration of camrelizumab was used until disease progression or unexpected toxicity. The dose and interval of TKIs allowed changes depending on toxicity and disease condition. Systematic therapy was suspended during the TACE procedure and resumed after TACE (7, 20).

### Follow Up

Multiphase enhanced CT or MRI was performed before treatment, 1-3 months after initial treatment, and every 2-3 months thereafter. Tumor response was evaluated according to the Modified Response Evaluation Criteria in Solid Tumors (mRECIST) (21). If disease progression (PD) was established at the first assessment, further investigation was conducted for possible hyperprogressive disease. Hyperprogression was defined as a progressive disease with an absolute increase in the tumor growth rate exceeding 50% per month (22), recorded as a delta tumor growth rate >50%.

To avoid interference from postembolization syndrome, treatment-related adverse events (AEs) were assessed at least 1 month after TACE. AEs were graded according to the National Cancer Institute Common Toxicity Criteria Adverse Events (CTCAE) version 5.0.

Barcelona Clinic Liver Cancer (BCLC) stage, Child–Pugh status, Eastern Cooperative Oncology Group performance status score (ECOG), and whole laboratory values within 1 month before treatment initiation and following treatment intervals were recorded.

The primary outcome was safety. Secondary outcomes included objective response rate (ORR), time to response (TTR), duration of response (DOR), disease control rate (DCR), time to progression (TTP), and progression-free survival (PFS). TTP was calculated from the first dose of ICI administration combined with TACE to radiological progression. If patients died without radiological progression, data were censored at the date of final imaging evaluation. PFS was calculated from the date of first ICI administration combined with TACE to radiological progression or death. If patients were still alive without radiological progression, data were censored at the cutoff day.

### Statistical Analysis

Kaplan–Meier analysis was used to estimate TTP and PFS, log-rank analysis was performed for comparison, and *a P* value ≤0.05 was considered statistically significant. Statistical analysis was performed using SPSS version 26 software (IBM, New York).

## Results

### Patients

**Figure 1:**
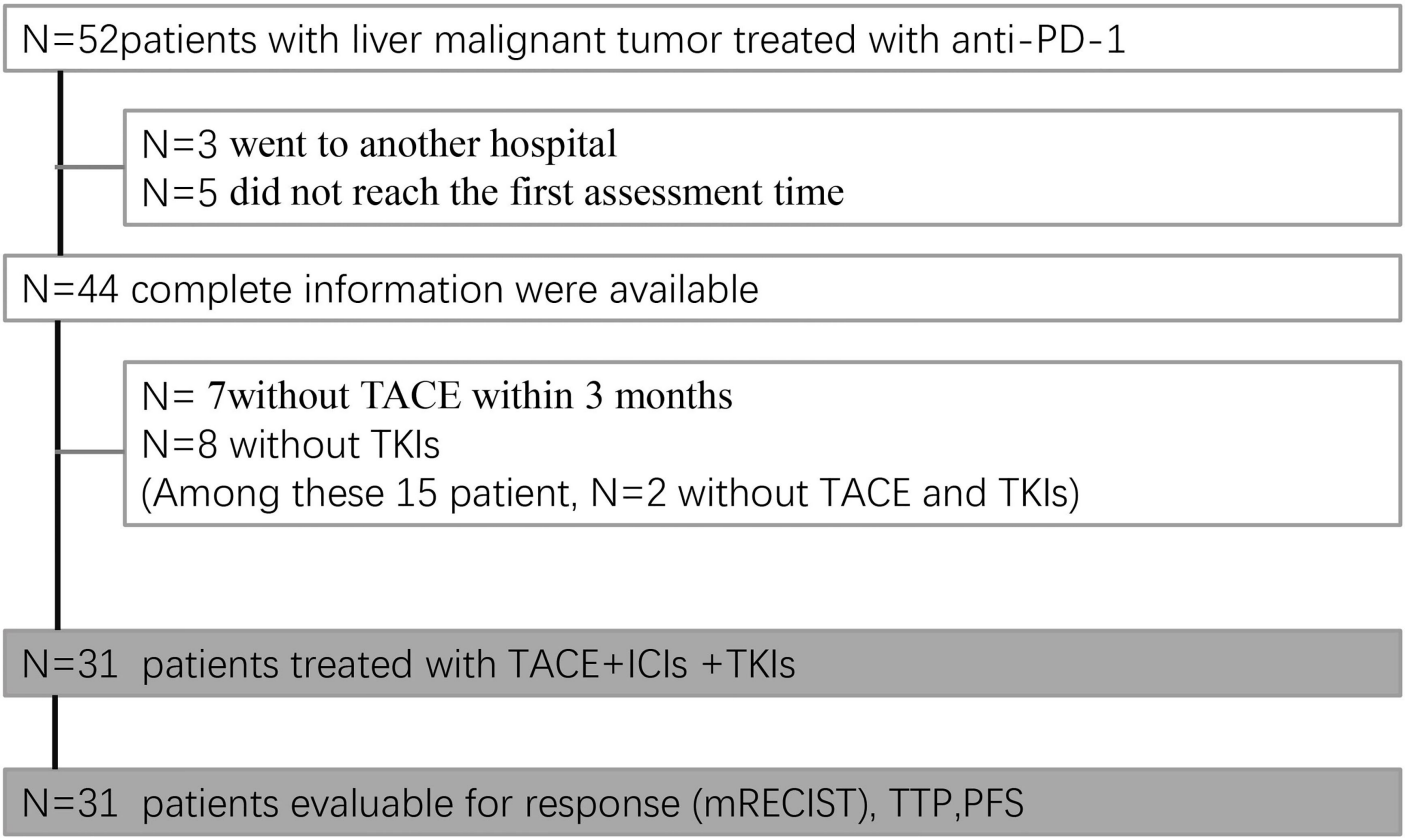
Patient flow chart.

From August 1, 2019, to July 31, 2020, a total of 52 patients received ICIs in our department. The date of data cutoff was October 31, 2020. Among 52 patients, 3 suspended medical care, 5 did not have follow-up imaging available, and the remaining 44 could be assessed. Seven patients did not undergo TACE within 3 months, and 8 patients did not receive TKIs. Among these 15 patients, 2 did not receive either TACE or TKIs; thus, 31 patients received the established triple combination therapy (TACE+ ICIs +TKIs) (**Figure 1**).

Of the 31 patients, 12 were diagnosed by surgery or biopsy, and 19 were clinically diagnosed. Patient characteristics are summarized in **Table 1**. Most of the disease etiology was hepatitis B (26/31, 83.9%). At the start of ICIs, most patients had a Child–Pugh A status (27/31, 87.1%) and ECOG performance status of 0 (20/31, 64.5%) or 1 (11/31, 35.5%). A quarter of all patients had AFP≥400 IU/ml (8/31, 25.8%). Two patients were classified as BCLC-A, 18 as BCLC-B and 11 as BCLC-C. Nineteen patients had multiple lesions, and nearly half of the patients had lesions distributed through the bilateral hepatic lobes (12/31, 38.7%). Cumulative targeted lesions were 69 mm (range, 21-170 mm) according to mRECIST.

**Table 1:** Baseline characteristics in 31 patients (mean age 57.5 y ± 9.4).

Characteristic	Number	percentage
**Sex**		
** female**	6	19.4
** male**	25	80.6
**Etiology**		
** Hepatitis B**	26	83.9
** others**	5	16.1
**Child-pugh class**		
** A**	27	87.1
** B**	4	12.9
**AFP**		
** <400 (IU/ml)**	23	74.2
** ≥400 (IU/ml)**	8	25.8
**Tumor distribution**		
** one hepatic lobe**	19	51.3
** two hepatic lobe**	12	38.7
**Tumor number**		
** <3**	12	38.7
** ≥3**	19	61.3
**BCLC**		
** A**	2	6.5
** B**	18	58
** C**	11	35.5
**ECOG PS**		
** 0**	20	64.5
** 1**	11	35.5
**Immunotherapy**		
** First-line**	19	61.3
** Second-line**	8	25.8
** Third-line**	3	9.7
** Fouth-line**	1	3.2

BCLC, Barcelona Clinic Liver Cancer; ECOG PS, Easten Cooperative Oncology Group Performance Status.

**Figure 2:**
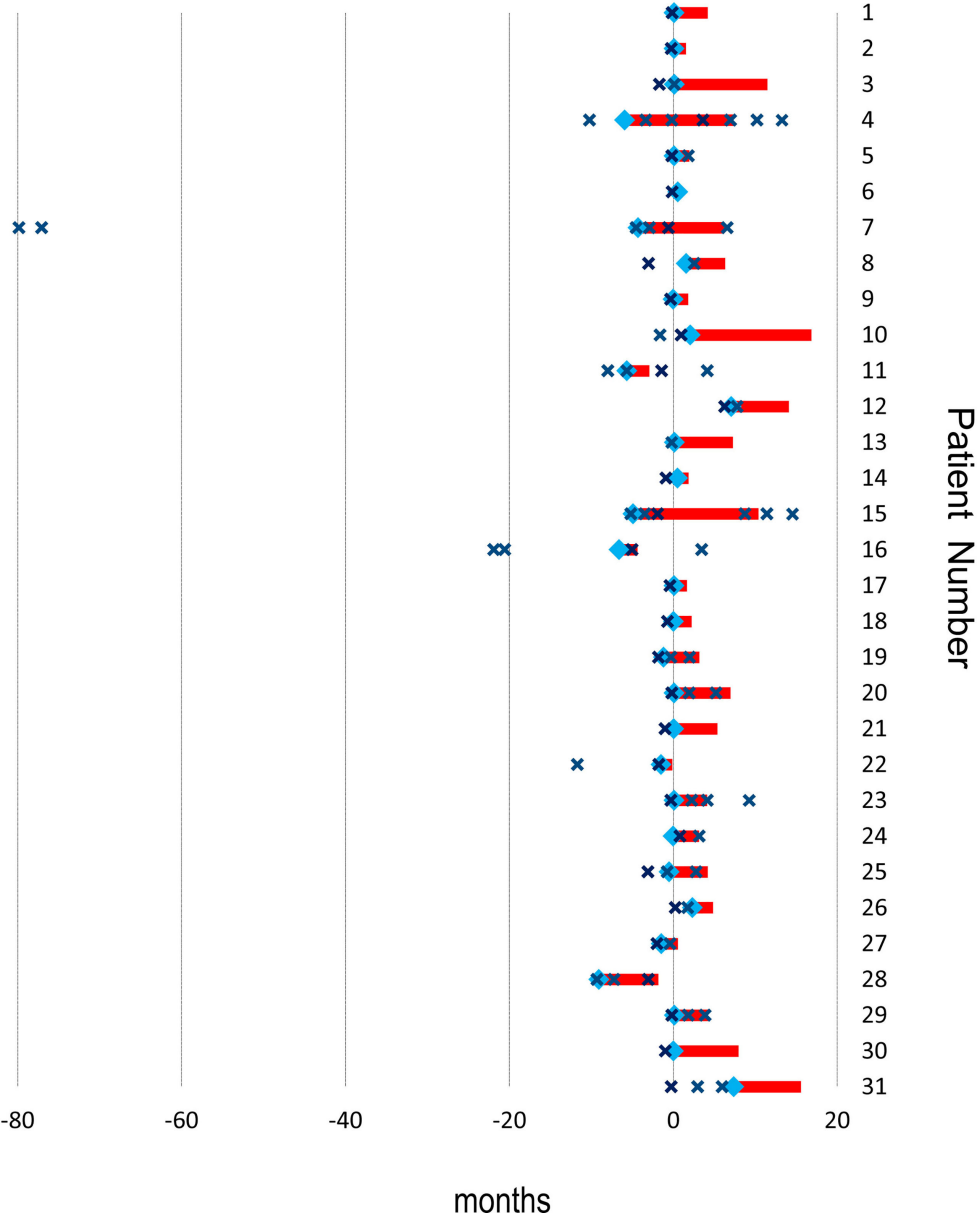
Treatment sequence. Red bar: duration of immunotherapy; ×: time of TACE; blue square; initial time of TKIs. X-axis: months. Y-axis: Patients Nr.

The treatment sequences are shown in **Figure 2**. The median number of TACE procedures per patient was 2.55 (range, 1-8), and the median number of TACE procedures combined with ICIs was 2.06 (range, 1-6). Five patients received initial ICIs prior to TACE, and 26 patients received ICIs after TACE. The median time of ICI immunotherapy was 5.68 cycles (range, 1-18 cycles). Fourteen patients received sorafenib, and 17 patients received lenvatinib. Ten patients received TKIs prior to ICIs, and 21 patients received TKIs after ICIs.

Twelve patients had prior treatment, including TACE (n=4), radioactive seed ^125^I implantation (n=2), ablation (n=1), ICI immunotherapy (n=1), TACE and ablation (n=3), TACE and ablation and ^125^I implantation (n=1). ICIs were used as first-, second-, third-, or fourth-line treatments in 19 (61%), 8 (26%), 3 (10%), and 1 (3%) patients, respectively.

### Safety

Twenty-nine (93%) patients experienced at least one adverse event (AE) during the treatment (**Table 2**). An incidence greater than 10% includes reactive capillary endothelial proliferation (RCCEP, n=15; 48.4%), total bilirubin increase (n=13; 41.9%),TSH increase (n=13; 41.9%), hypothyroidism (n=9; 29%), alanine aminotransferase increase (n=8; 25.8%), aspartate aminotransferase increase (n=7; 22.6%), proteinuria (n=7; 22.6%), hypertension (n=6; 19.4%), cardiac troponin (n=5; 16.1%), hyperthyroidism (n=4; 12.9%), hand-foot syndrome (n=4; 12.9%), and diarrhea (n=4; 12.9%). Four (12.9%) patients developed AEs of higher grade (grade≥3), total bilirubin increase (n=2;6.5%), proteinuria (n=1;3.2%), and platelet count decrease (n=1;3.2%). No patient died due to AEs. One patient discontinued camrelizumab due to grade 3 proteinuria.

**Table 2:** Adverse events.

event	Anygrade n (%)	grade 3 or 4 n (%)
Proteinuria	7 (22.6)	1 (3.2)
Aspartate aminotransferase increase	7 (22.6)	0
Hypertension	6 (19.4)	0
Diarrhea	4 (12.4)	0
Decreased appetite	3 (9.7)	0
Pyrexia	3 (9.7)	0
Alanine aminotransferase increase	8 (25.8)	0
Blood Total bilirubin increase	13 (41.9)	2 (6.5)
Nausea	2 (6.5)	0
Platelet count decrease	1 (3.2)	1 (3.2)
reactive capillary endothelial proliferation	15 (48.4)	0
hoarse voice	2 (6.5)	0
hand-foot syndrome	4 (12.9)	0
TSH	13 (41.9)	0
hyperthyroidism	4 (12.9)	0
hypothyroidism	9 (29)	0
cardiac troponin	5 (16.1)	0
myoglobin	2 (6.5)	0
BNP	2 (6.5)	0
hyperglycemia	1 (3.2)	0
hypophysitis	3 (9.7)	0
musculoskeletal pain	1 (3.2)	0

### Efficacy

**Figure 3:**
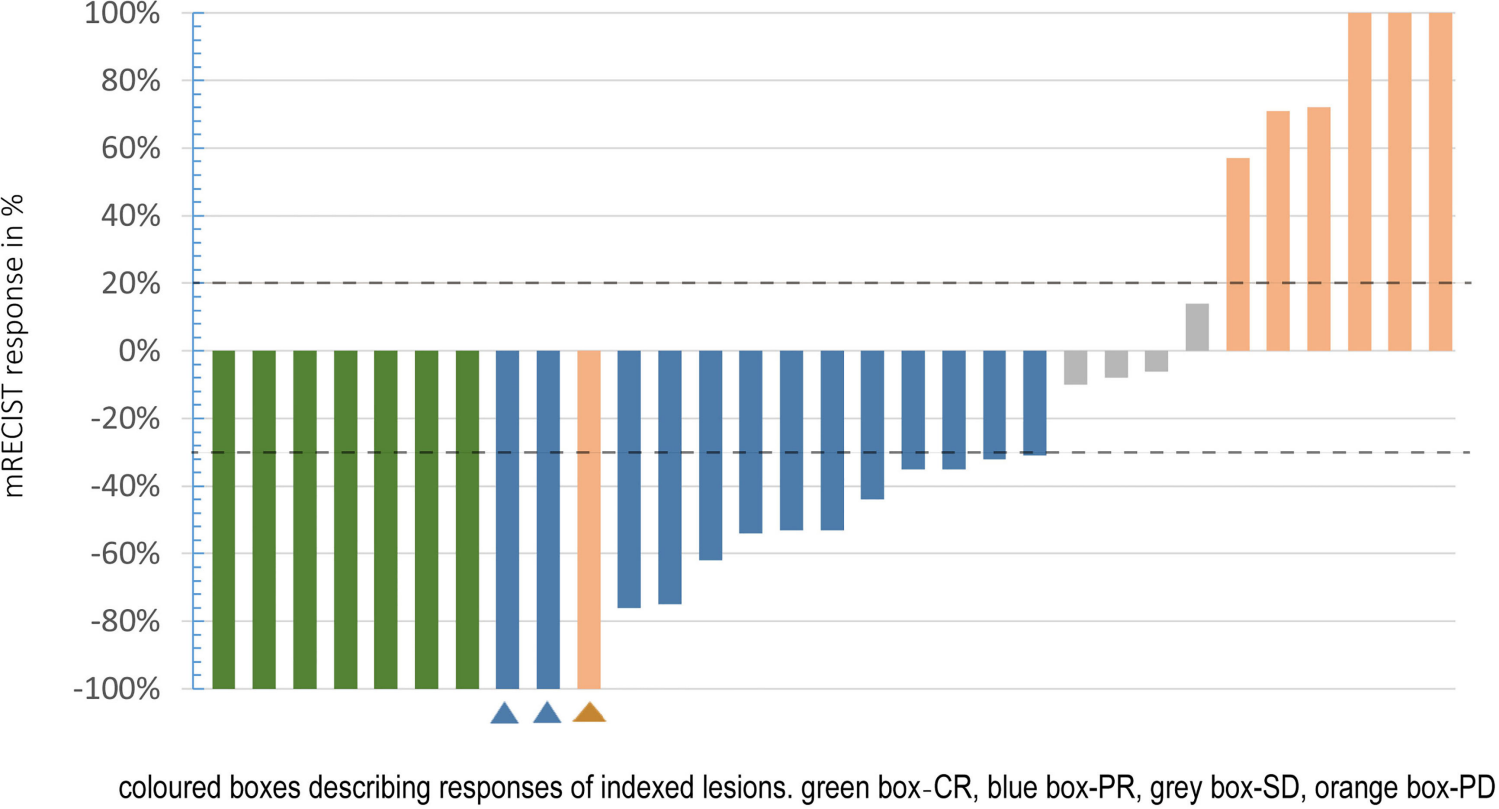
Waterfall plot shows the response of targeted tumor (mRECIST). Green box: CR; blue box: PR; grey box: SD; orange box: PD. Dashed lines indicate thresholds for PR (less than 30%) or PD (>20%). 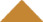
patient had vertebral metastasis; 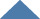
Patients had nontargeted tumor survival.

At the time of review, all patients had at least one follow-up image for radiological tumor response assessment. Of the targeted tumors, 10 patients achieved a complete response; among them, 2 patients had nontargeted tumor survival, resulting in a partial response, and 1 patient had vertebral metastasis, resulting in progressive disease. As of the best treatment response, 7 patients had a complete response (23%), and 13 patients achieved a partial response (41.5%). Thus, the objective response rate (ORR) was 64.5%. Four patients had stable disease (12.9%), resulting in a disease control rate (DCR) of 77.4% (**Table 3** and **Figure 3**).

**Table 3:** Radiological response according to mRECIST and clinical efficacy.

	Number	percentage (%)
best response		
CR	7	22.6
PR	13	41.9
SD	4	12.9
PD	7	22.6
ORR (CR+PR)	20	64.5
DCR (CR+PR+SD)	24	77.4
TTP, median (95% CI)	6.5 (3.5-11)	
PFS, median (95% CI)	8.5 (3.5-NE)	

**Figure 4:**
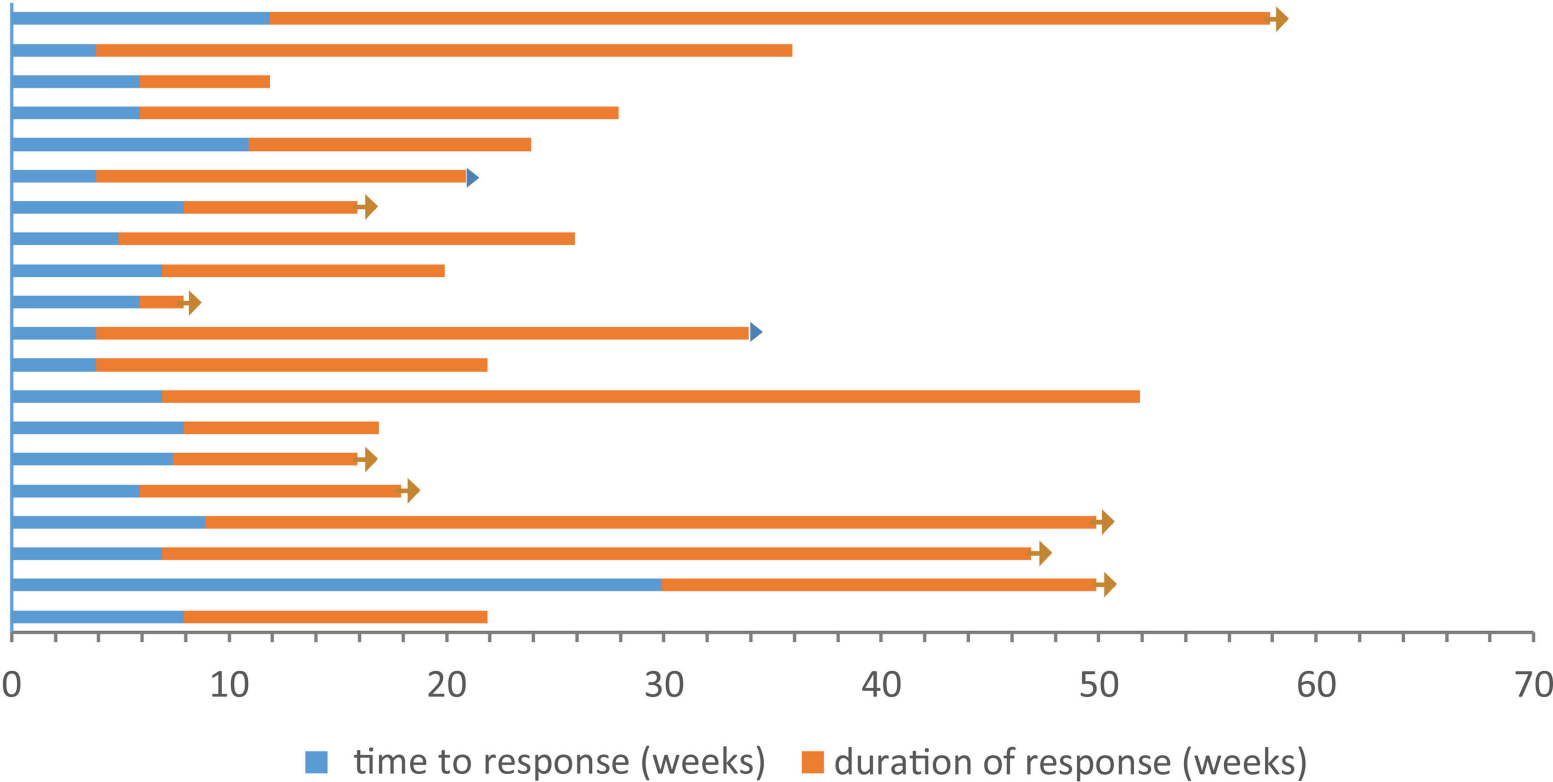
Response and duration for 20 patients with best overall response of ORR (CR or PR), unit: weeks. 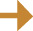
response ongoing at last assessment 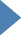
death at last assessment.

A total of 20 patients achieved an objective response, and the median time to response was 7 weeks (range, 4-30 weeks). At the time of the last radiographic assessment, 10 patients finally achieved PD. The duration of response was 17.5 weeks (range, 2-46 weeks) (**Figure 4**). There were two patients with hyperprogressive disease: one patient survived 5.5 months, and the other patient survived only 4 months.

**Figure 5:**
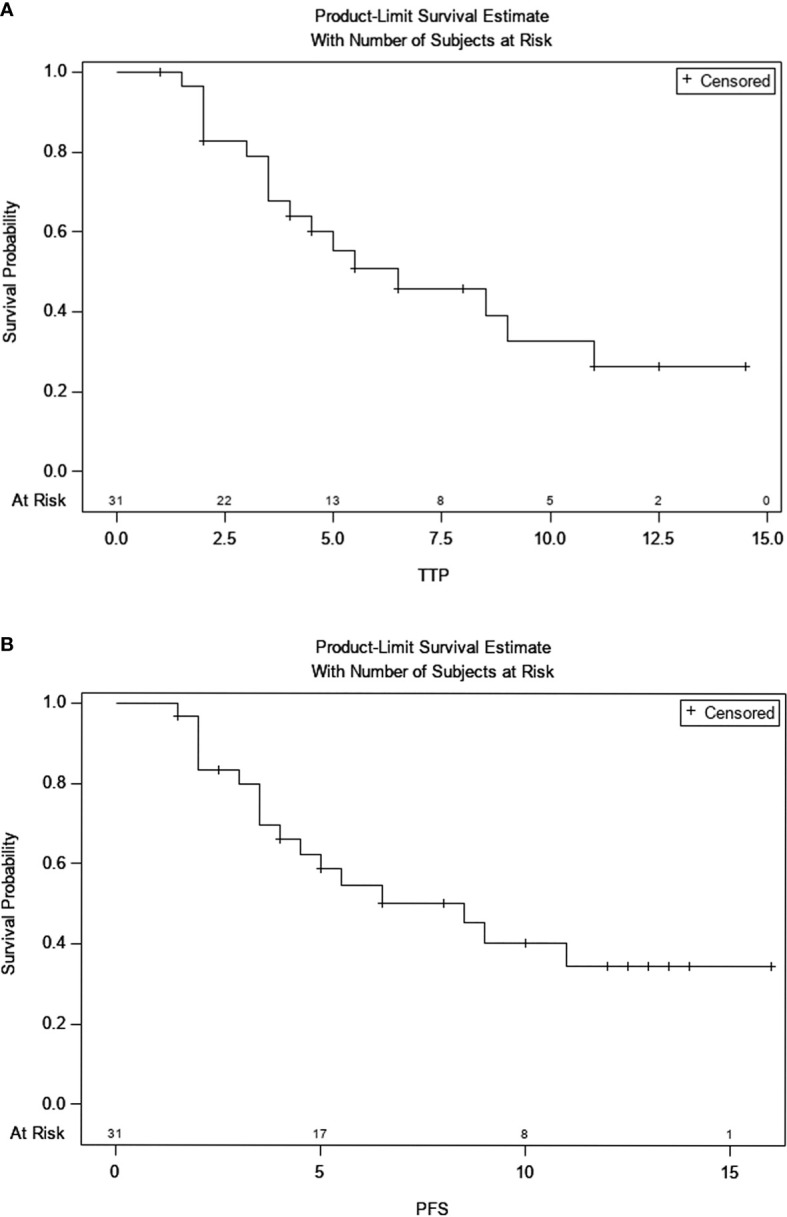
**(A)** Kaplan-Meier curve of time to progression; **(B)** Kaplan-Meier curve of progression free survival.

At the date of cutoff, the median duration of follow-up was 9 months. Twenty-one patients were still alive, and 10 patients continued to receive immunotherapy. ICIs were discontinued mainly due to disease progression (n=7) or adverse events (n=1) or other treatments (ablation, n=1; sorafenib, n=2). The subsequent treatments after disease progression were ablation (n=4) and radioactive seed ^125^I implantation (n=3). Overall, TTP was 6.5 months (95% CI, 3.5-11), and PFS was 8.5 months (95% CI, 3.5-NE) (**Figure 5**). There were no significant differences in PFS between BCLC stage (p=0.056) and C-P grade (p=0.175), between the sequence of ICIs and TKIs (p=0.332) or between the sequence of ICIs and TACE (p=0.932). Similarly, there were no significant differences in TTP based on the same subgroup (**Table 4**).

**Table 4:** Subgroup analysis of clinical efficacy.

		Chi-Square	P
BCLC			
(B vs C)	PFS	5.7553	0.0563
	TTP	5.9026	0.0523
Child-Pugh_Class			
(A vs B)	PFS	1.8386	0.1751
	TTP	0.7547	0.385
sequence of ICIs and TKIs			
ICIs-pre vs TKIs-pre	PFS	0.9403	0.3322
	TTP	0.2295	0.6319
sequence of ICIs and TACE			
ICIs-pre vs TACE-pre	PFS	0.0071	0.9329
	TTP	0.0428	0.8362

## Discussion

This study found that twenty-nine (93%) patients experienced at least one treatment-related adverse event (AE), 12.9% patients (n=4) experienced treatment-related grade 3 AEs, and one patient discontinued ICIs due to grade 3 proteinuria and no treatment-related death. The most common AE was RCCEP (n=15; 48.4%), which is a typical AE related to camrelizumab. The incidence of RCCEP has been reported to be approximately 67%-76.7% when treated alone with HCC or esophageal carcinoma (12, 23) and can be alleviated by apatinib (24). Thus, this relatively low incidence of RCCEP may have contributed to the combination of TKIs. Twelve percent of patients (n=4) experienced treatment-related grade 3 AEs, and one patient discontinued immunotherapy due to grade 3 proteinuria. This is comparable with two other reports with locoregional therapy combined with ICIs (9, 10), which reported 19% (5/26) and 13% (4/29) of patients with grade 3 AEs and no treatment-related death. Recently, ESMO reported another triple therapy combining HAIC+ lenvatinib+toripalimab (anti-PD-1) (25). In that cohort, grade 3 AEs were neutropenia (8.5%), thrombocytopenia (5.6%), and nausea (5.6%). Altogether, the triple combination therapy (TACE+ICIs+TKIs) appears to be safe and feasible.

The result of this reach reported an ORR of 64.5% and a DCR of 77.4%, which exceed the similar results of immunotherapy trials to date. A phase 2 clinical trial of ICI monotherapy demonstrated an ORR of 14.7% for camrelizumab (12); when camrelizumab was combined with apatinib (13), an ORR of 50% (8/16) and a PFS of 5.8 months were reported. In a randomized phase 3 trial, atezolizumab (anti-PD-L1) plus bevacizumab (anti-VEGF antibody) achieved an ORR of 33% and a DCR of 73.6% (14). Compared to combinations with different systemic therapies, locoregional therapy plus systemic therapy seems to further improve efficacy. Locoregional therapy, including TACE, can induce ‘immunogenic cell death’ by releasing tumor antigens, which may facilitate antitumor immunity (26, 27).

When compared to other retrospective studies of TACE plus ICIs in the real world, this cohort still found comparable clinical efficacy, with a TTP of 6.5 months (95% CI, 3.5-11) and a PFS of 8.5 months (95% CI, 3.5-NE). Zhan et al. (9) examined 26 patients with intermediate to advanced HCC who underwent ICI immunotherapy within 90 days of radioembolization and reported a higher ORR of 77% and TTP and PFS of 5.7 months. Marinelli et al. (10) investigated TACE or transarterial radioembolization (TARE) with nivolumab in 29 patients with BCLC B or C HCC. They reported an ORR of 45% in the first months, and in the TACE cohort, TTP was 4.3 months. They had nearly similar subjects (BCLC B or C HCC) and the number of cases as our cohort. Although comparisons between different studies should be made with caution, the data of this study indicated a longer PFS, which might have been due to the additional administration of TKIs. TACE can aggravate the hypoxic microenvironment of residual viable tumors. Subsequent angiogenesis occurs by proangiogenic cytokines, such as VEGF. Hypoxia and overexpression of VEGF lead to an immunosuppressive microenvironment (4). Preclinical studies have shown that lower doses of antitumor angiogenesis (anti-VEGFR2 antibody or TKIs) may reprogram the tumor immunosuppressive microenvironment through vascular normalization (28, 29). Tumor vascular normalization alleviates hypoxia and stimulates T lymphocyte infiltration, therefore enhancing the efficacy of immunotherapy (30). Interestingly, tumor vascular normalization could be conversely promoted by ICI therapy, and dual ICI/anti-VEGFR2 therapy further enhanced the efficacy of immunotherapy in an HCC model (31). This efficacy of the combination of ICIs with TKIs has been proven by several clinical trials (13, 14). As such, the rationale is clear to combine TACE with ICIs and TKIs: TACE alleviates tumor burden and facilitates antitumor immunity, and the combination of ICIs with TKIs can treat residual viable tumors through the mechanism mentioned above.

Lai et al. (25) also reported a triple combination therapy of HAIC+ lenvatinib+toripalimab (anti-PD-1), which is the most similar research to the current study. They included 71 patients with advanced HCC and demonstrated an ORR of 59.2%, DCR of 90.1%, and PFS of 11.1 months. It is worth noting that, at the cutoff date of the current study, 21 out of 31 patients were still alive, and the clinical efficacy should be continued. In the CheckMate 040 study (32), among the nivolumab (NIVO) + ipilimumab (IPI) + cabozantinib (CABO) subgroup of patients with advanced HCC (n = 35), the ORR was 26%, DCR was 83%, and PFS was 6.8 months. However, in the NIVO + IPI+ CABO arm, grade 3-4 AEs were observed in 71% of patients (n=25).

During this research, the dose and interval of TKIs were not fixed and could be determined according to the specific situation. This is because a low dose of TKIs promotes normalization of the tumor vasculature (33). Interrupted dosing with sorafenib, with the aim of reducing overlapping toxicity, has also shown better OS and local response rates than TACE alone (34).

There are several limitations in this study. Due to the retrospective nature of the study, the heterogeneity of treatments could not be well controlled, and prior treatments might have affected the final clinical outcomes. The relatively small patient population also limited patient stratification, and there were no statistically significant differences in TTP and PFS between the TACE sequence and ICIs or ICIs and TKIs. This result should be cautiously interpreted because of the underpowering of this study. In addition, the lack of a control group in this study limited the generalizability of the results.

In conclusion, this triple combination therapy appears to have an acceptable safety profile and considerable efficacy in patients with uHCC. The results of this study might promote prospective studies in the future.

## Data Availability Statement

The raw data supporting the conclusions of this article will be made available by the authors, without undue reservation.

## Ethics Statement

The studies involving human participants were reviewed and approved by the Institutional Review Board of the First Affiliated Hospital of Soochow University (Suzhou, Jiangsu Province, China). The patients/participants provided their written informed consent to participate in this study.

## Author Contributions

X-LZ and W-SW contributed to the study concept and design. FY, JY, WX, WC-L, SZ, and YY contributed to the acquisition of clinical data. FY wrote the first draft of the manuscript. X-LZ, W-SW, BY-Z, and JS supervised and oversaw the study. H-PS contributed to the statistical analysis. All authors contributed to the article and approved the submitted version.

## Funding

This study was supported by the National Natural Science Foundation of China (81771945), Jiangsu Province Key Health Personnel Program (ZDRCA2016038), Key R&D Program(Social Development) Project of Jiangsu Province (BE2021648). Funding source had no involvement in the financial support for the conduct of the research and preparation of the article.

## Conflict of Interest

The authors declare that the research was conducted in the absence of any commercial or financial relationships that could be construed as a potential conflict of interest.

## Publisher’s Note

All claims expressed in this article are solely those of the authors and do not necessarily represent those of their affiliated organizations, or those of the publisher, the editors and the reviewers. Any product that may be evaluated in this article, or claim that may be made by its manufacturer, is not guaranteed or endorsed by the publisher.

## Abbreviations

HCC, hepatocellular carcinoma; TACE, transarterial chemoembolization; ICIs, Immune checkpoint inhibitors; TKIs, tyrosine kinase inhibitors; uHCC, unresectable hepatocellular carcinoma; AEs, adverse events; CTCAE, the National Cancer Institute Common Toxicity Criteria Adverse Events; mRECIST, Modified Response Evaluation Criteria in Solid Tumor; ECOG, Eastern Cooperative Oncology Group performance status score; ORR, objective response rate, TTR, time to response; DOR, duration of response; DCR, disease control rate; TTP, time to progression; PFS, progression free survival; VEGF, vascular endothelial growth factor; RCTs, randomized controlled trials; MDT, multi-disciplinary Treatment; BCLC, Barcelona Clinic Liver Cancer; AFP, alpha-fetoprotein.
